# Monochromatic Light Pollution Exacerbates High-Fat Diet-Induced Adipocytic Hypertrophy in Mice

**DOI:** 10.3390/cells11233808

**Published:** 2022-11-28

**Authors:** Qingyun Guan, Yixuan Li, Zixu Wang, Jing Cao, Yulan Dong, Fazheng Ren, Yaoxing Chen

**Affiliations:** 1 Neurobiology Laboratory, College of Veterinary Medicine, China Agricultural University, Haidian, Beijing 100193, China; 2 Key Laboratory of Precision Nutrition and Food Quality, Key Laboratory of Functional Dairy, Ministry of Education, Beijing Laboratory of Food Quality and Safety, Department of Nutrition and Health, China Agricultural University, Beijing 100083, China

**Keywords:** monochromatic light pollution, adipose hypertrophy, high-fat diet, circadian clocks, corticosterone

## Abstract

Light pollution worldwide promotes the progression of obesity, which is widely considered a consequence of circadian rhythm disruptions. However, the role of environmental light wavelength in mammalian obesity is not fully understood. Herein, mice fed a normal chow diet (NCD) or a high-fat diet (HFD) were exposed to daytime white (WL), blue (BL), green (GL), and red light (RL) for 8 weeks. Compared with WL and RL, BL significantly increased weight gain and white adipose tissue (WAT) weight, and it disrupted glucose homeostasis in mice fed with HFD but not NCD. The analysis of WAT found that BL significantly aggravated HFD-induced WAT hypertrophy, with a decrease in IL-10 and an increase in NLRP3, p-P65, p-IκB, TLR4, Cd36, Chrebp, Srebp-1c, Fasn, and Cpt1β relative to WL or RL. More interestingly, BL upregulated the expression of circadian clocks in the WAT, including Clock, Bmal1, Per1, Cry1, Cry2, Rorα, Rev-erbα, and Rev-erbβ compared with WL or RL. However, most of the changes had no statistical difference between BL and GL. Mechanistically, BL significantly increased plasma corticosterone (CORT) levels and glucocorticoid receptors in the WAT, which may account for the changes in circadian clocks. Further, in vitro study confirmed that CORT treatment did promote the expression of circadian clocks in 3T3-L1 cells, accompanied by an increase in Chrebp, Cd36, Hsp90, P23, NLRP3, and p-P65. Thus, daily BL, rather than RL exposure-induced CORT elevation, may drive changes in the WAT circadian clocks, ultimately exacerbating lipid dysmetabolism and adipocytic hypertrophy in the HFD-fed mice.

## 1. Introduction

With the acceleration of urbanization, the massive introduction of artificial light at night (LAN) has blurred the normal time division of day and night, making light pollution a potential global health risk factor [1,2]. Meanwhile, growing exposure to light from electronic products has also exacerbated the progress of light pollution. In modern society, people are exposed to intelligent device screens, such as mobile phones and computers, for a long time during the daytime, which emits blue light (BL) that is harmful to health. The unreasonable or irregular use of artificial light may be responsible for bringing terrible environmental disasters to the health of humans and animals.

Interestingly, the influence of light pollution on metabolic homeostasis has raised increasing attention in recent years [3]. Irregular light exposure has been proven to be a hazardous factor for obesity [4,5,6], as it promotes hepatic steatosis [7,8], increases fasting blood glucose (FBG), and induces insulin resistance (IR) [9,10]. The effects of ambient light exposure on metabolic homeostasis depend on light period, intensity, and wavelengths [11]. As reported, the extent to which dim LAN (dLAN) and constant light (LL) exposure dysregulate metabolisms differs [12]. Exposure to LAN has an intensity-dependent acute detrimental effect on glucose metabolism [13]. However, studies on the link between wavelength and metabolism are limited. The existing findings have not fully revealed the role of light wavelength, barely diving into the physiological mechanisms involved in the light wavelength’s impact at the lipid metabolism level.

There are currently more than 1.9 billion adults worldwide who are overweight or obese. These staggering numbers continue to climb, posing inestimable health and economic burdens [14]. The increased white adipose tissue (WAT) in obesity is a highly active but dysfunctional metabolic organ, which is one of the main stages of complex mechanisms that underlie the pathogenesis of obesity, namely low-grade inflammation [15]. On the contrary, thermogenic brown adipose tissue (BAT) contributes to weight loss due to its abundant mitochondria. Nevertheless, BAT dysfunction leads to obesity, a process linked to the “whitening” of the tissue [16]. Moreover, abnormal changes in genes involved in lipid synthesis, transport, and oxidation can affect lipid accumulation in adipose and lead to WAT hypertrophy or BAT whitening [17]. 

Notably, obesity exacerbated by light pollution is now commonly linked to the disruption of the circadian clock. In the core cycle of the circadian clock, the main components include Circadian Locomotor Output Cycles Kaput (CLOCK), Brain and Muscle Arnt-like Protein 1 (BMAL1), Cryptochrome (CRY1, CRY2), and Period (PER1, PER2, PER3). Additional feedback loops include nuclear receptors retinoic acid-related orphan receptors (RORs), REV-ERBs., etc., to maintain the robustness of the clock system [18]. The circadian system is important for energy metabolism, which is synchronized mainly through the standard light-dark (LD) cycle [19]. On the contrary, irregular light is transmitted to the suprachiasmatic nucleus (SCN) via intrinsic photosensitive retinal ganglion cells (ipRGCs), disturbing the central circadian rhythm and driving changes in peripheral circadian clocks, which is likely to be mediated by hormone signals such as melatonin and corticosterone (CORT) [18]. In the adipose tissue, biological clock genes in the adipose tissue regulate the proliferation and differentiation of adipocytes, lipid metabolism, and endocrine [20,21].

Although some knowledge has been gained about the relationship between light wavelength and obesity, the effect and mechanism on the adipose tissue remain unknown, especially for diurnal light exposure. Therefore, this study aims to explore the effect and potential pathways of long-term exposure to diurnal monochromatic lights on obesity in mice from the perspective of the adipose tissue, thus providing a brand-new theoretical reference for the prevention or treatment of obesity.

## 2. Materials and Methods

### 2.1. Animal Treatment and Light Exposure

The experiment is based on the “Guidelines for the Care and Use of Laboratory Animals” issued by the Animal Welfare Committee of the Agricultural Research Organization, China Agricultural University (Approval No.AW18079102-1-2). Six-week-old male C57BL/6 mice (Charles River Co., Ltd., Beijing, China) were raised under optimum conditions (at a temperature of 21 ± 1 °C, relative humidity of 50 ± 10%, 14-h:10-h LD cycle) and obtained food and water freely. The mice were firstly housed under white light (WL, 400–700 nm) and a normal chow diet (NCD, 4% of energy from lipid, Charles River Co. Ltd., Beijing, China) for 1-week adaption. Then, the mice were randomly divided into (1) mice exposed to WL fed on NCD (WN) or on a high-fat diet (HFD, 45% of energy from lipid, Beijing HFK Bioscience Co., Ltd., Beijing, China) (WF), (2) mice exposed to BL (peak at 444 nm) fed on NCD (BN) or HFD (BF), (3) mice exposed to green light (GL, peak at 528 nm) fed on NCD (GN) or HFD (GF), and (4) mice exposed to red light (RL, peak at 624 nm) fed on NCD (RN) or HFD (RF). The lights were powered by a light-emitting diode system (Zhongshan Junsheng Lighting Technology Co., Ltd. Zhongshan, China) with a constant intensity of 150 lx under 14-h:10-h LD cycle (lights on from 7:00 AM to 9:00 PM). The lights’ parameters are shown in Table 1. During the experiment, animal body weight, food intake, and water drinking were recorded. After 8 weeks, the plasma and adipose tissues were harvested for the following experiments.

### 2.2. Glucose and Insulin Tolerance Test

After 12 h overnight, mice were intraperitoneally injected with glucose (1 g/kg body weight, Sigma, St. Louis, MO, USA) for a glucose tolerance test (GTT). Blood samples from the tip of the tail were measured at 0, 15, 30, 60, 90, and 120 min after glucose injection by a portable glucose monitor (Aike, Lingrui, Hangzhou, China). The area under the curve (AUC) was calculated by Graphpad prism (version 9.4, GraphPad Software Inc., San Diego, CA, USA). For the insulin tolerance test (ITT), mice were fasted overnight for 6 h to inject insulin (0.75 IU/kg, Novolin R, Novo Nordisk, Denmark), and their blood glucose was measured as described.

### 2.3. Commercial Kits Detection

The concentration of CORT, melatonin, and noradrenalin in plasma samples and the concentrations of inflammatory factors (IL-6 and IL-10) in the WAT were detected by a competitive enzyme-linked immunosorbent assay (Uscn Life Science, Inc., Wuhan, China). Commercial kits (Jiancheng Institute of Biotechnology, Nanjing, China) were used to determine the plasma levels of total cholesterol (TC) and triglyceride (TG). All the tests were carried out according to the manufacturer’s instructions.

### 2.4. Histology Staining

The harvested epididymis (Epi)-WAT and BAT were maintained in 4% formalin for 48 h, followed by a series of alcohol dehydrations, and finally embedded in paraffin. Epi-WAT and BAT (5 μm) were sectioned using a semiautomatic rotary microtome, stained with Hematoxylin and Eosindye (H&E), and observed microscopically (BX51, Olympus, Tokyo, Japan). The density of adipocytes in WAT was measured as the ratio of the number of cells to the total area of the section field of view. Measurement of mean adipocyte area was found using automated Image-pro plus software (Media Cybernetics, Inc., Rockville, MD, USA). The WAT was examined from six animals in each group.

### 2.5. Quantitative Real Time (RT)-PCR Analysis

We used a previously described method [22]. In short, total mRNA was first extracted from the Epi-WAT, BAT, and cell, followed by reverse transcription to obtain cDNA. Then, RT-PCR experiments were conducted via designed gene primers and demonstrated the expression level of each gene with glyceraldehyde-3-phosphate dehydrogenase (Gapdh) as an internal control. The experiments were repeated in triplicate. The primers used are shown in Table 2.

### 2.6. Western Blot Analysis

Epi-WAT and cell proteins were extracted and performed western blot detection according to the previously described method [22]. The specific antibodies and related concentrations were as follows: NOD-like receptor thermal protein domain associated protein 3 (NLRP3, 1:1000; CST), p-P65 (1:1000; CST), p-IκB (1:1000; CST), toll-like receptor 4 (TLR4, 1:1000; Santa Cruz, Dallas, TX, USA), circadian locomotor output cycles kaput (CLOCK, 1:1000; Abcam, Cambridge, UK), brain and muscle arnt-like protein 1 (BMAL1, 1:1000; Santa Cruz, Dallas, TX, USA), glucocorticoid receptor (GR, 1:2000; Proteintech, Rosemont, IL, USA), and β-actin (1:8000; Proteintech, Rosemont, IL, USA) overnight at 4 °C. The membranes were washed with Tris-buffered saline Tween and incubated with horseradish peroxidase-conjugated goat anti-mouse/rabbit antibody (1:8000; CoWin Biotech Co., Inc., Cambridge, MA, USA). The target band values were normalized to those of β-actin. The results were based on three independent experiments.

### 2.7. Cell Culture and Treatment

3T3-L1 cell lines were maintained in Dulbecco’s modified Eagle’s medium supplemented with 10% fetal bovine serum and 4 mM l-glutamine + 100 U/mL penicillin + 100 µg/mL streptomycin + 4500 mg/L glucose, and cultured at 37 °C in a 95% air/5% CO_2_ humidity environment. Cells were plated at a density of 8 × 10^3^ cells/well in 96-well plates for 3-(4,5-dimethylthiazol-2-yl)-2,5-diphenyltetrazolium bromide (MTT) assay to observe the effect of CORT on cell’s viability. Briefly, cells were seeded and cultured in 96-well plates for 6 h. Subsequently, we replaced the complete medium with the basal medium and continued to culture for 12 h. Then, cells were treated with various concentrations (1 μM, 5 μM, 10 μM, 20 μM, and 30 μM) of CORT (MedChemExpress, Prinbceton, NJ, USA) for 24 h. Next, MTT solution (5 mg/mL in PBS, Sigma, St. Louis, MO, USA) was added and incubated for 4 h. Afterwards, the supernatant was removed and the crystals were dissolved with 150 μL of DMSO (Sigma, St. Louis, MO, USA). The absorbance at 490 nm was measured. The 3T3-L1 cells were then treated with 1 μM (L-CORT) and 10 μM (H-CORT) CORT respectively for the subsequent RT-PCR and WB assay.

### 2.8. Statistical Analysis

Data are expressed as the mean ± standard error and were analyzed using Graphpad Prism (version 9.4, GraphPad Software Inc., San Diego, CA, USA). One-way ANOVA was used to statistically analyze differences between groups. *p <* 0.05 was considered statistically significant.

## 3. Results

### 3.1. Effects of Monochromatic Light Exposure on Metabolic Disorders in Mice

**Figure 1:**
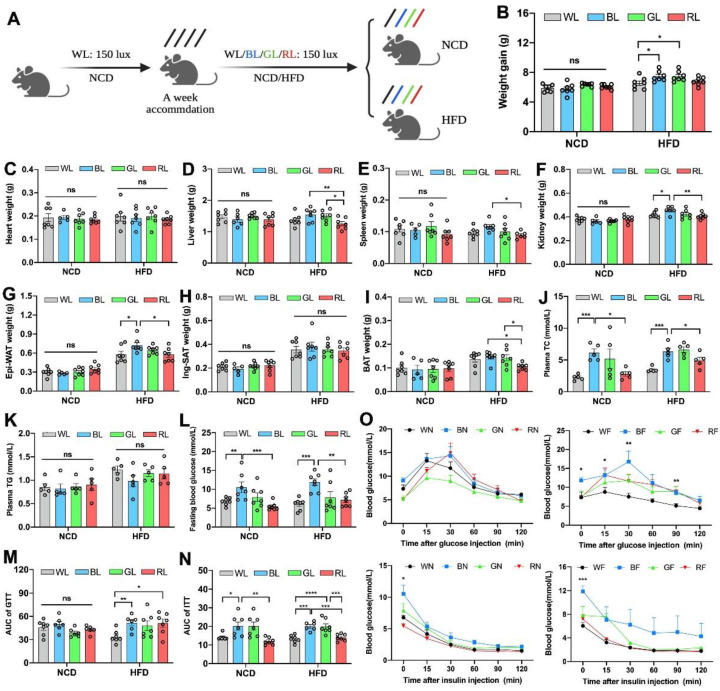
Influences of monochromatic light exposure on metabolic disorders in mice. (**A**) Schematic diagram of animal experiments. (**B**) Body weight gain (*n* = 7). (**C**–**I**) Weights of the heart, liver, spleen, kidney, Epi-WAT, Ing-WAT, and BAT (*n* = 7). (**J**,**K**) Plasma TC and TG concentrations (*n* = 5). (**L**) Fasting blood glucose level (*n* = 7). (**M**–**O**) GTT and ITT curve and relevant AUC (*n* = 7). The circles represent the number of samples. The results are presented as the means ± SEM. * *p* < 0.05, ** *p* < 0.01, *** *p* < 0.001, **** *p* < 0.0001.

To determine the effect of different wavelengths of light exposure on body weight and obesity, 7-week-old mice fed with NCD or HFD were respectively raised under WL, BL, GL, and RL exposure for 8 weeks (Figure 1A). As shown in Figure 1B, there were no significant changes in weight gain between light colors under NCD feeding (*p* > 0.05). However, under HFD feeding, BL increased weight gain by 14.2% compared with WL (*p* < 0.05), while having an insignificant change with GL (*p* > 0.05). Consistent with the changes in body weight gain, light colors did not change the weights of the heart (Figure 1C), liver (Figure 1D), spleen (Figure 1E), kidney (Figure 1F), Epi-WAT (Figure 1G), inguinal subcutaneous adipose tissue (Ing-SAT, Figure 1H), and BAT (Figure 1I) under NCD feeding. However, in the HFD-fed groups, BL increased the weights of most of these organs. Besides, plasma total cholesterol (TC) was significantly elevated by BL compared to WL and RL under both NCD and HFD (*p* < 0.05, Figure 1J), while there was no statistical difference in triglycerides (TG, *p* > 0.05, Figure 1K). To examine the effect on glucose homeostasis, we measured the level of fasting blood glucose (FBG) and found that BL exposure accentuated HFD-induced increases in FBG compared with WL and RL, by 31.3% and 114.6% (*p* < 0.01, Figure 1L), respectively. Results of the GTT and ITT (Figure 1M–O) and AUC values further showed that BL sharpened insulin resistance and insulin resistance impaired by HFD compared with WL and RL (*p* < 0.01).

### 3.2. Effects of Monochromatic Light Exposure on WAT Hypertrophy and Inflammation in Mice

**Figure 2:**
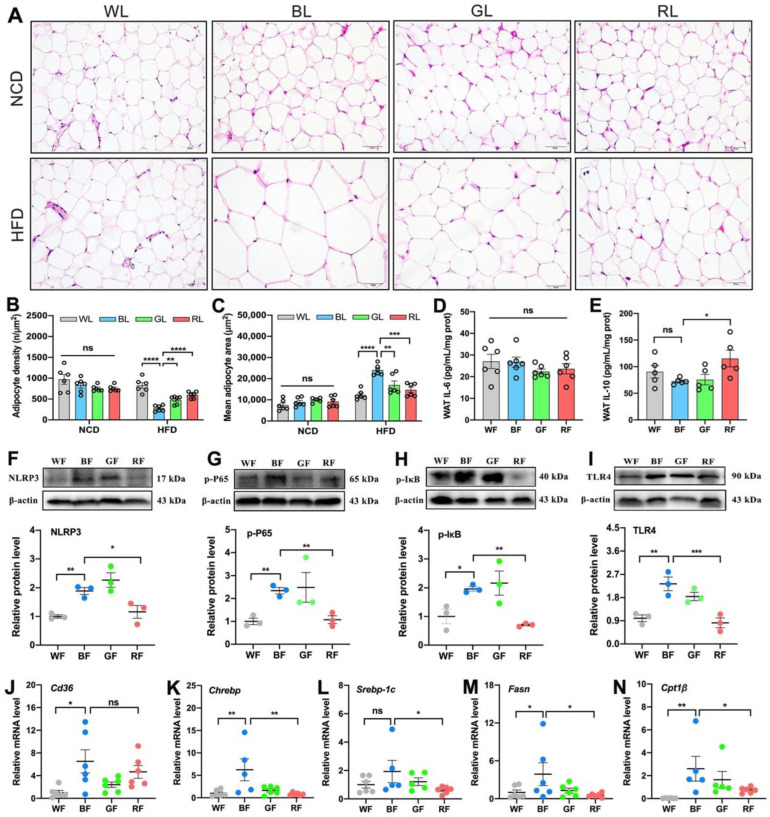
Influences of monochromatic light exposure on WAT hypertrophy and inflammation in mice. (**A**) H&E staining of the Epi-WAT (scale: 50 μm). (**B**) Adipocyte density (*n* = 6). (**C**) Mean adipocyte size (*n* = 6). (**D**,**E**) Concentrations of IL-6 and IL-10 in the Epi-WAT (*n* = 6). (**F**–**I**) Relative protein expression levels of NLRP3, p-P65, p-IκB, and TLR4 in the Epi-WAT (*n* = 3). (**J**–**N**) Relative mRNA expression levels of Cd36, Chrebp, Srebp-1c, Fasn, and Cpt1β (*n* = 6). The circles represent the number of samples. The results are presented as the means ± SEM. * *p* < 0.05, ** *p* < 0.01, *** *p* < 0.001, **** *p* < 0.0001.

As obesity and the increased Epi-WAT weight were previously observed, we further measured the size of adipocytes gained from the Epi-WAT sections via H&E staining (Figure 2A). Consistently, there was also no significant change in the adipocyte density (*p* > 0.05, Figure 2B) and mean adipocyte size (*p* > 0.05, Figure 2C) between light colors under NCD feeding. Nevertheless, BL reduced the adipocyte density (*p* < 0.001) and enlarged the mean adipocyte size (*p* < 0.001) compared with WL, GL, and RL under HFD. Based on the insignificant differences in NCD, we focused on HFD feeding in the following study. As shown in Figure 2D–I, BL significantly inhibited the levels of IL-10 in the Epi-WAT as compared with RL (*p* < 0.05), though no changes were exerted in the level of IL-6 (*p* > 0.05). In parallel, BL consistently increased relative protein levels of NLRP3 (*p* < 0.01, Figure 2F), p-P65 (*p* < 0.01, Figure 2G), p-IκB (*p* < 0.05, Figure 2H), and TLR4 (*p* < 0.05, Figure 2I) relative to WL and RL. Likewise, there was no statistical difference between BL and GL at the level of inflammation (*p* > 0.05). Next, we further examined the expression of genes related to lipid metabolism in the Epi-WAT (Figure 2J–N), including those involved in fatty acid transporters (Cd36), de novo lipogenesis (carbohydrate response element binding protein, Chrebp; sterol regulatory element binding protein-1C, Srebp-1c; fatty acid synthase, Fasn), and fatty acid (FA) β-oxidation (carnitine palmitoyl transferase 1β, Cpt1β). Compared with WL or RL, BL significantly increased the levels of Cd36, Chrebp, Srebp-1c, Fasn, and Cpt1β (*p* < 0.05), while GL did not (*p* > 0.05).

### 3.3. Effects of Monochromatic Light Exposure on the Expression of Circadian Clock in the WAT

**Figure 3:**
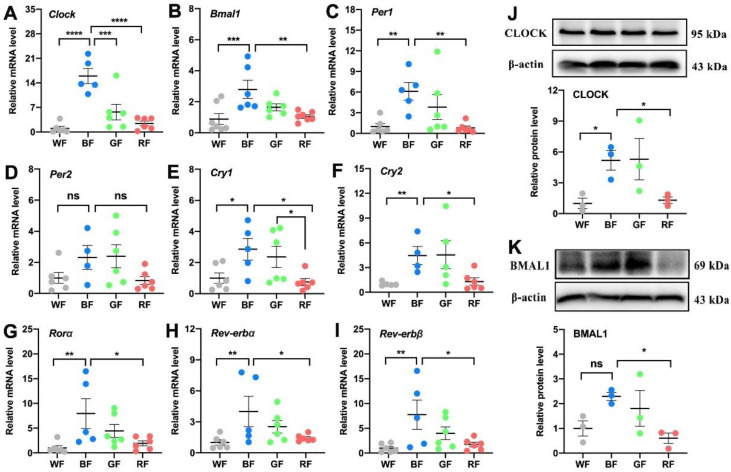
Influences of monochromatic light exposure on the expression of circadian genes in the WAT. (**A**–**I**) Relative mRNA expression levels of Clock, Bmal1, Per1, Per2, Cry1, Cry2, Rorα, Rev-erbα, and Rev-erbβ in the Epi-WAT (*n* = 6). (**J**,**K**) Relative protein expression levels of CLOCK and BMAL1 in the Epi-WAT (*n* = 3). The results are presented as the means ± SEM. * *p* < 0.05, ** *p* < 0.01, *** *p* < 0.001, **** *p* < 0.0001.

Since the metabolic abnormalities affected by light pollution may be controlled by biological clocks, we then examined the expression of circadian-related molecules in the WAT, including transcripts encoding Clock, Bmal1, Per1, Per2, Cry1, Cry2, Rorα, Rev-erbα, and Rev-erbβ. Compared with WL or RL, BL consistently increased the mRNA expression levels of Clock (*p* < 0.001, Figure 3A), Bmal1 (*p* < 0.01, Figure 3B), Per1 (*p* < 0.01, Figure 3C), Cry1 (*p* < 0.05, Figure 3E), Cry2 (*p* < 0.05, Figure 3F), Rorα (*p* < 0.05, Figure 3G), Rev-erbα (*p* < 0.05, Figure 3H), and Rev-erbβ (*p* < 0.05, Figure 3I), except for Per2 (*p* > 0.05, Figure 3D). In parallel, BL exposure elevated the expression levels of CLOCK and BMAL1 compared to WL or RL at the protein level (*p* < 0.05, Figure 3J,K).

### 3.4. Effects of Monochromatic Light Exposure on BAT Whitening and Expression Levels of Circadian Genes in the BAT

**Figure 4:**
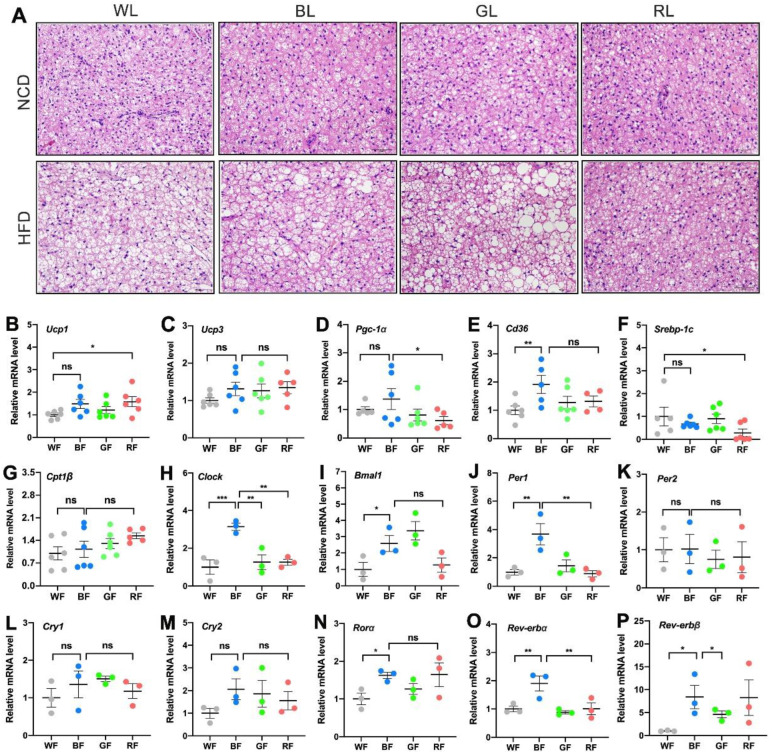
Influences of monochromatic light exposure on BAT whitening and expression of circadian genes in BAT. (**A**) H&E staining of BAT (scale: 50 μm). (**B**–**G**) Relative mRNA expression levels of genes involved in lipid metabolism in the BAT, including Ucp1, Ucp3, Pgc-1α, Cd36, Srebp-1c, Cpt1β (*n* = 6). (**H**–**P**) Relative mRNA expression levels of Clock, Bmal1, Per1, Per2, Cry1, Cry2, Rorα, Rev-erbα, and Rev-erbβ in the BAT (*n* = 3). The results are presented as the means ± SEM. * *p* < 0.05, ** *p* < 0.01, *** *p* < 0.001.

The proper function of BAT is critical to the fight against obesity. As shown in Figure 4A, HFD-fed mice exhibited markedly enhanced lipid accumulation (i.e., “whitening”) under WL, BL, and GL, except for RL exposure. Then we examined the expression of genes related to lipid metabolism in the BAT. Compared to WL, BL significantly increased the mRNA expression of uncoupling protein (Ucp) 1 (*p* < 0.05, Figure 4B) and Cd36 (*p* < 0.01, Figure 4E), while RL significantly increased the expression of Ucp1 (*p* < 0.05). There were no statistical differences in the expression of Ucp3 (*p* > 0.05, Figure 4C) and Cpt1β (*p* > 0.05, Figure 4G) among the groups. Furthermore, consistent with the result in the WAT, BL increased the expression of clock genes in the BAT compared with WL or RL, including Clock (*p* < 0.01, Figure 4H), Bmal1 (*p* < 0.05, Figure 4I), Per1 (*p* < 0.01, Figure 4J), Rorα (*p* < 0.05, Figure 4N), Rev-erbα (*p* < 0.01, Figure 4O), and Rev-erbβ (*p* < 0.05, Figure 4P), except Per2 (*p* > 0.05, Figure 4K), Cry1 (*p* > 0.05, Figure 4L), Cry2 (*p* > 0.05, Figure 4M).

### 3.5. The Role of CORT in Interference with the Adipose Circadian Clock

**Figure 5:**
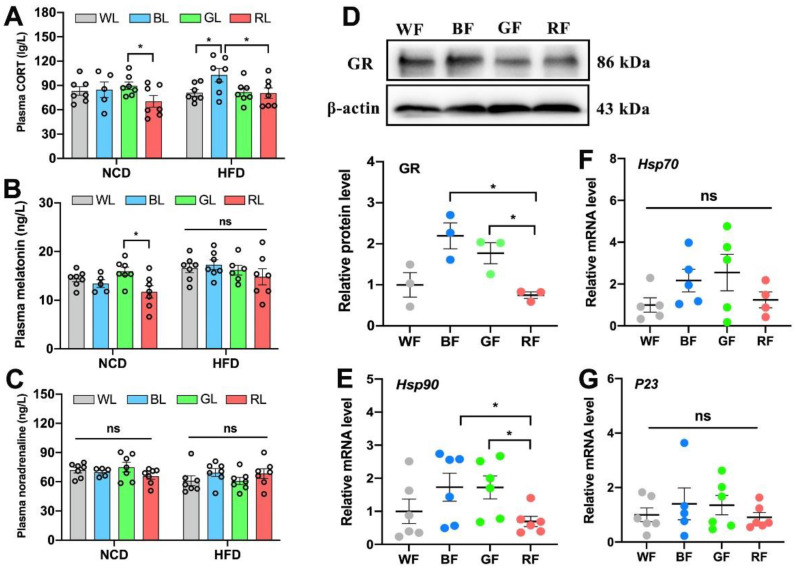
Influences of monochromatic light exposure on CORT concentrations. (**A**–**C**) Plasma concentrations of CORT, melatonin, and noradrenaline (*n* = 7). (**D**) Relative protein expression level of GR in the Epi-WAT (*n* = 3). (**E**–**G**) Relative mRNA expression levels of Hsp90, Hsp70, and P23 in the Epi-WAT (*n* = 6). The circles represent the number of samples. The results are presented as the means ± SEM. * *p* < 0.05.

Finally, to investigate the mechanism by which light exposure influenced the adipose circadian clock, we focused on the hormonal pathways of output and feedback of the central circadian system. As shown in Figure 5A, the concentration of plasma CORT in the HFD-fed group was significantly higher by 21.3% and 27.6% in BL compared with WL and RL (*p* < 0.05), respectively. However, the concentrations of plasma melatonin (*p* > 0.05, Figure 5B) and noradrenaline (*p* > 0.05, Figure 5C) were not statistically significant among light colors under HFD feeding. Then, we investigated whether different wavelengths affect the expression level of GR in the WAT. Consistently, the expression of GR in BL was 65.9% more than that of RL (*p* < 0.05, Figure 5D). We then examined whether GR transport was also affected. Compared with RL, BL significantly increased the expression of Hsp90 mRNA (*p* < 0.05, Figure 5E), while there was no statistically significant difference in the expressions of Hsp70 (*p* > 0.05, Figure 5F) and P23 (*p* > 0.05, Figure 5G) mRNA. Therefore, BL increased plasma CORT levels and enhanced GR synthesis and transport in the HFD-fed mice.

**Figure 6:**
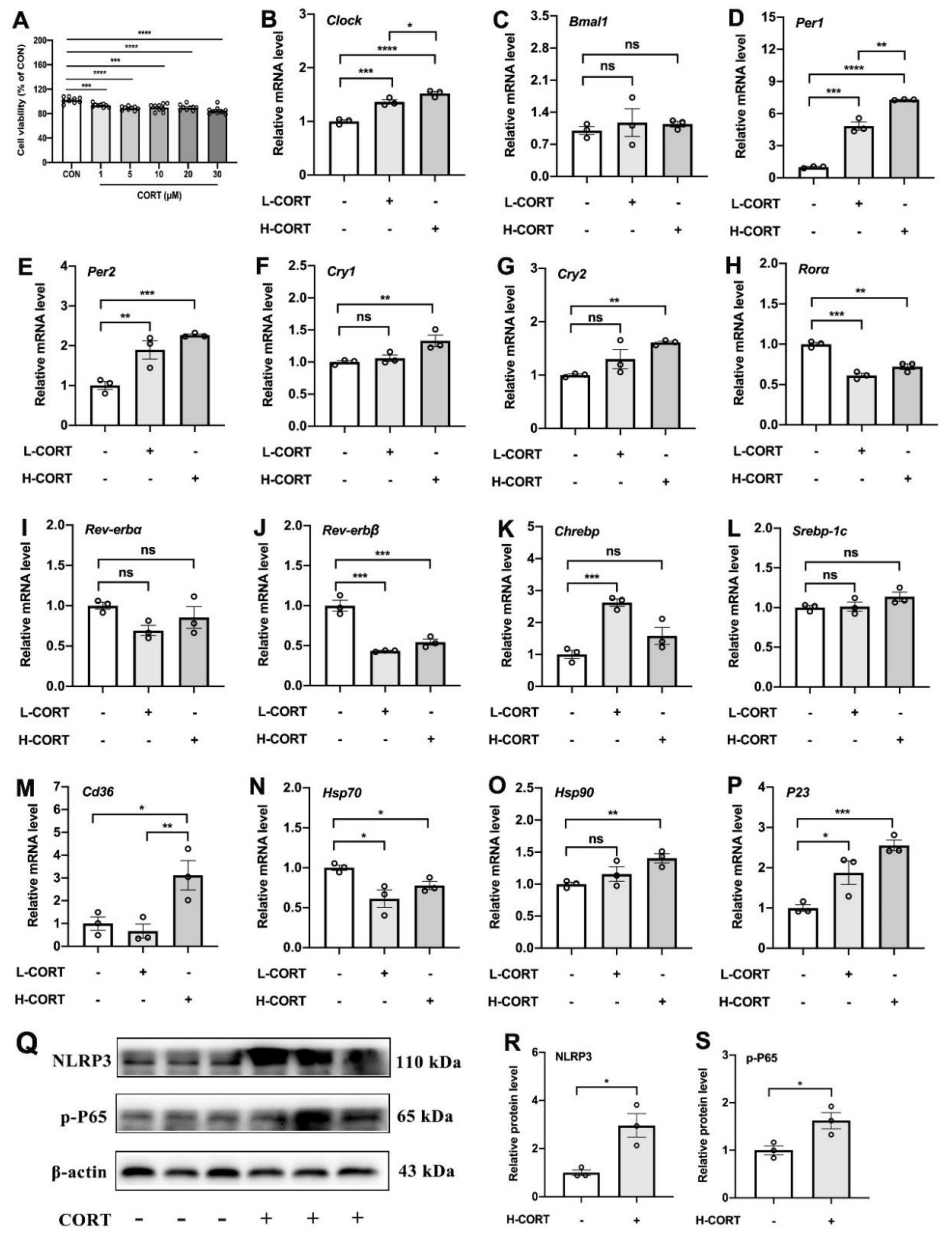
The role of CORT in interference with the adipose circadian rhythms. (**A**) Cell viability (% of CON). (**B**–**P**) Relative mRNA expression levels of Clock, Bmal1, Per1, Per2, Cry1, Cry2, Rorα, Rev-erbα, Rev-erbβ, Chrebp, Srebp-1c, Cd36, Hsp70, Hsp90, and P23 in 3T3-L1 cells treated by CORT (*n* = 3). (**Q**–**S**) Relative protein levels of NLRP3 and p-P65 (*n* = 3). The circles represent the number of samples. The results are presented as the means ± SEM. * *p <* 0.05, ** *p <* 0.01, *** *p <* 0.001, **** *p <* 0.0001.

As CORT is one of the output and feedback signals of the central circadian system that may drive changes in the peripheral biological clock, we hypothesized that adipose circadian clocks may be affected by the elevated CORT. To test our hypothesis, we added CORT to treat 3T3-L1 cell lines. As shown in Figure 6A, CORT promoted cell death in a dose-dependent manner (*p* < 0.001). Compared with the control group, both L-CORT and H-CORT increased the mRNA expression levels of Clock (Figure 6B), Per1 (Figure 6D), and Per2 (Figure 6E). Moreover, CORT increased the mRNA expression levels of Cry1 (Figure 6F) and Cry2 (Figure 6G). Besides, CORT treatment significantly increased the mRNA expression levels of Chrebp (Figure 6K), Cd36 (Figure 6M), Hsp90 (Figure 6O), and P23 (Figure 6P), as well as increased levels of NLRP3 and p-P65 proteins *(p* < 0.05, Figure 6Q–S).

## 4. Discussion

The wide application of artificial lighting technology and electronic products inevitably exposes organisms to light pollution. The effects of light pollution on metabolic diseases such as obesity [23], impaired glucose tolerance [24], and non-alcoholic fatty liver disease [25] are partially understood, but most of this knowledge is based on nighttime WL exposure. There is a growing force to uncover the role and ways in which light wavelengths in the development of obesity, especially daytime exposure.

Previous studies suggested that BL exposure at night disrupted glucose metabolism [24,26], and continuous BL exposure aggravated HFD-induced obesity in mice [22]. Besides, daytime GL exposure promoted the development of hepatic steatosis and pancreatic dysfunction [27]. However, there is no direct evidence of the effect of light colors on adipose tissues, which displayed the most immediate role in obesity. Herein, we constructed a mouse model exposed to different diurnal light wavelengths under an NCD or HFD for 8 weeks to investigate the influence of the diurnal spectrum on adipose tissues. Our results showed that under the NCD feeding, there were no statistical differences in body weight gain, adipose tissue weight, and WAT expansion under different light colors. Nevertheless, these changes could be exaggerated in the case of HFD feeding. Compared with WL or RL, BL exposure increased body weight gain, WAT’s weight, and disrupted glucose homeostasis. Further analysis of WAT found that BL significantly promoted WAT hypertrophy and inflammatory response in the HFD-fed mice, accompanied by a reduction in IL-10 and an increase in the expression of NLRP3, p-P65, p-IκB, and TLR4 in BL relative to WL and RL. Adipocytic hypertrophy is related to the abnormal increase in lipid accumulation. Lipid metabolism in the body involves a series of processes. Activation of transcription factors such as Chrebp and Srebp-1c contribute to FA synthesis, which is transported by FA transporters such as Cd36 [28]. Our results showed that BL increased the mRNA level of Cd36, Chrebp, and Cpt1β in the WAT, which may further promote lipid accumulation and hypertrophy in the adipocyte. In parallel, BL promoted BAT whitening induced by HFD compared with RL. However, there was no significant difference between BL and GL. Altogether, BL may play a more critical role in adipocytic hypertrophy than WL or RL in HFD-fed mice, and nutritional signaling may be involved in the role of synergistic light wavelengths in mammalian lipid dysmetabolism.

To a large extent, metabolic disorders induced by irregular light have to do with circadian clock disturbance. A normal circadian system is essential for maintaining lipid metabolic homeostasis [29]. For instance, Clock mutant mice have a diminished daily feeding rhythm and promote obesity [30]. Deletion of Bmal1 may induce higher or lower body fat [31,32]. Normalization of the circadian clock in adipose tissue controls processes such as lipogenesis and lipolysis, as many key enzymes involved in lipolysis and lipogenesis are directly regulated by the circadian clocks [33]. In our study, BL exposure significantly increased the expression levels of clock genes in the WAT compared with WL or RL, including Clock, Bmal1, Per1, Cry1, Cry2, Rorα, Rer-erbα, and Rer-erbβ. The same was true in BAT, i.e., BL increased the expression of Clock, Per1, and Rer-erbα compared with RL. Therefore, we hypothesized that changes in clock genes in the WAT may be related to abnormalities in genes involved in lipid metabolism. In addition, differences among light wavelengths are most likely due to the short wavelength, perceived as BL, being the strongest synchronizer of the circadian system, which synchronizes most biological and psychological rhythms internally [34]. However, the circadian system is less sensitive to RL because RL cannot activate melanopsin-containing retinal ganglion cells that project to the SCN [18,35]. Hence, BL instead of RL exposure interfered with the adipose circadian rhythms in the HFD-fed mice, which, to some extent, may support the above results.

**Figure 7:**
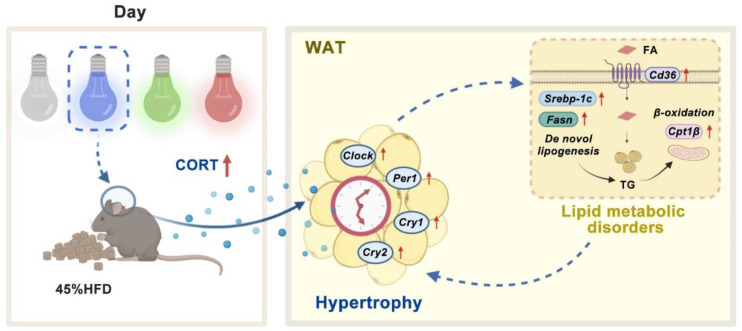
A schematic of our proposed model. Effects of long daytime monochromatic light exposure on adipose hypertrophy in mice fed with HFD. BL exposure increases the level of plasma CORT, which disturbs the circadian clocks in the WAT. Changes in circadian clocks may further regulate the process of lipid metabolism and ultimately lead to adipose hypertrophy. CORT, corticosterone; FA, fatty acid; HFD, high-fat diet; TG, triglyceride; WAT, white adipose tissue.

As thus, light color-induced disruptions in the adipose circadian clock caught further attention. Hormonal signals, including melatonin and CORT, are one of the key pathways that SCN controlled the peripheral clock [18,36]. Hence, we inferred that light-induced changes in the adipose circadian clock were driven by altered central circadian clock output hormonal signals. Through measuring plasma melatonin and CORT levels, it was found that BL significantly increased plasma CORT compared with WL and RL, while there were no significant effects on melatonin. In addition, the BL exposure group was accompanied by an increase in GR synthesis and transport in the WAT. Therefore, CORT may participate in the process of driving the biological clock changes of the WAT. To further verify the direct effect of CORT on circadian clocks, we treated 3T3-L1 cells with CORT. Both low and high concentrations of CORT increased the overall expression levels of the circadian clock in cells, including Clock, Per1, and Per2, and high CORT concentration increased the mRNA expression levels of Cry1 and Cry2. CORT treatment also increased the mRNA levels of Chrebp, Cd36, Hsp90, and P23 mRNA, and protein levels of NLRP3 and p-P65. Consistent in vivo and in vitro results suggest that CORT does play a vital role in disrupting the circadian clocks of adipose tissues, which may further contribute to lipid metabolic disorder and inflammatory response. In conclusion, in our model, long-term BL exposure was likely to exacerbate adipose hypertrophy induced by HFD in mice, which may be related to the change of adipose clock induced by CORT (Figure 7).

Notably, there are still some limitations within this study. Firstly, the nocturnal rodents used here may not fully mimic the effects of daytime spectral effects in humans, as nocturnal rodents and diurnal humans may have different physiological responses to light exposure. Secondly, the changes in the peripheral clocks we examined were only at one point in time rather than at various Zeitgeber times. Furthermore, we focused on the role of CORT in interfering with the adipose clock, yet whether the same is true in BL exposure has not been fully established. Little is known about how diurnal monochromatic light affects CORT, which is another shortcoming of our study.

## 5. Conclusions

In general, our results highlight the critical role of BL exposure in shaping obesity in HFD-fed mice. Long-term diurnal BL exposure exacerbated adipocyte hypertrophy, most likely due to its changes in the adipose circadian clock affected by the increased CORT. The role of monochromatic light pollution on adipose tissue opens a new avenue for interventions against ambient monochromatic light pollution and obesity.

## Figures and Tables

**Table 1:** Light parameters.

Items	Light Exposure			
	WL	BL	GL	RL
Light wavelength (nm)	400–700	Peak at 444	Peak at 528	Peak at 624
Light intensity (lux)	150	150	150	150
Light: dark cycle (h)	14:10	14:10	14:10	14:10

**Table 2:** Sequences of primers used for RT-PCR.

Gene	Primer Sequence (5′ to 3′)		Product Size	Accession
Cd36	F: GTGCAAAACCCAGATGACGT	R: TCCAACAGACAGTGAAGGCT	180	NM_001159558.1
Chrebp	F: GTGTGTGGTTTCGTGACCC	R: CACTTGTGGTATTCGCGCATC	128	NM_001359237.1
Srebp-1c	F: ATCGCAAACAAGCTGACCTG	R: AGATCCAGGTTTGAGGTGGG	115	NM_001388389.1
Fasn	F: TCCTGGAACGAGAACACGATCT	R: GAGACGTGTCACTCCTGGACTTG	138	NM_007988.3
Cpt1β	F: GGCACCTCTTCTGCCTTTAC	R: TTTGGGTCAAACATGCAGAT	136	NM_009948.2
Clock	F: ATGGTGTTTACCGTAAGCTGTAG	R: CTCGCGTTACCAGGAAGCAT	197	XM_011249402.3
Bmal1	F: CAGAGCCGGAGCAGGAAAAATAGGT	R: CAGGGGGAGGCGTACTTGTGATGT	128	NM_001374642.1
Per1	F: CGGATTGTCTATATTTCGGAGCA	R: TGGGCAGTCGAGATGGTGTA	142	NM_001159367.2
Per2	F: GAAAGCTGTCACCACCATAGAA	R: AACTCGCACTTCCTTTTCAGG	186	NM_011066.3
Cry1	F: CACTGGTTCCGAAAGGGACTC	R: CTGAAGCAAAAATCGCCACCT	153	NM_007771.3
Cry2	F: CACTGGTTCCGCAAAGGACTA	R: CCACGGGTCGAGGATGTAGA	102	NM_009963.4
Rorα	F: TCCAAATCCCACCTGGAAAC	R: GGAAGGTCTGCCACGTTATCTG	70	NM_001289916.1
Rve-erbα	F: TACATTGGCTCTAGTGGCTCC	R: CAGTAGGTGATGGTGGGAAGTA	127	NM_145434.4
Rve-erbβ	F: GGAAACACTCATCCGTGCACTA	R: ATCGAAGATCTGGCAACTTTAGAA	101	NM_001145425.2
Ucp1	F: TAAGCCGGCTGAGATCTTGT	R: GGCCTCTACGACTCAGTCCA	84	NM_009463.3
Ucp3	F: CTGCACCGCCAGATGAGTTT	R: ATCATGGCTTGAAATCGGACC	191	NM_009464.3
Pgc-1α	F: TATGGAGTGACATAGAGTGTGCT	R: CCACTTCAATCCACCCAGAAAG	134	NM_008904.3
Hsp70	F: CGGTGCCCGCCTACTTC	R: TCCTTCTTGTGCTTCCTCTTGA	322	NM_005346.6
Hsp90	F: ACGAGGAAGAGAAGAAGAAAATGG	R: GCAGGGTGAAGACACAAGCC	131	NM_001271971.2
P23	F: ATGCGTTTGGAGAAGGACAGA	R: CAGGGATGAAGTGATGGTGAG	210	NM_001289785.1
Gapdh	F: CCGAGAATGGGAAGCTTGTC	R: TTCTCGTGGTTCACACCCATC	232	NM_001289726.1

F = forward primer; R = reverse primer.

## Data Availability

Data are contained within the article.

## References

[1] Gaston K.J., Visser M.E., Hölker F. (2015). The biological impacts of artificial light at night: The research challenge. Philos. Trans. R. Soc. Lond. B Biol. Sci..

[2] Batra T., Malik I., Kumar V. (2019). Illuminated night alters behaviour and negatively affects physiology and metabolism in diurnal zebra finches. Environ. Pollut..

[3] Guan Q., Wang Z., Cao J., Dong Y., Chen Y. (2022). The role of light pollution in mammalian metabolic homeostasis and its potential interventions: A critical review. Environ. Pollut..

[4] Zhang D., Jones R.R., Powell-Wiley T.M., Jia P., James P., Xiao Q. (2020). A large prospective investigation of outdoor light at night and obesity in the NIH-AARP Diet and Health Study. Environ. Health.

[5] Esaki Y., Obayashi K., Saeki K., Fujita K., Iwata N., Kitajima T. (2021). Bedroom light exposure at night and obesity in individuals with bipolar disorder: A cross-sectional analysis of the APPLE cohort. Physiol. Behav..

[6] Lin L.Z., Zeng X.W., Deb B., Tabet M., Xu S.L., Wu Q.Z., Zhou Y., Ma H.M., Chen D.H., Chen G.B. (2022). Outdoor light at night, overweight, and obesity in school-aged children and adolescents. Environ. Pollut..

[7] Borck P., Batista T., Vettorazzi J., Soares G., Lubaczeuski C., Guan D., Boschero A., Vieira E., Lazar M., Carneiro E. (2018). Nighttime light exposure enhances Rev-erbα-targeting microRNAs and contributes to hepatic steatosis. Metab. Clin. Exp..

[8] Yue F., Xia K., Wei L., Xing L., Wu S., Shi Y., Lam S.M., Shui G., Xiang X., Russell R. (2020). Effects of constant light exposure on sphingolipidomics and progression of NASH in high-fat-fed rats. J. Gastroenterol. Hepatol..

[9] Borck P.C., Rickli S., Vettorazzi J.F., Batista T.M., Boschero A.C., Vieira E., Carneiro E.M. (2021). Effect of nighttime light exposure on glucose metabolism in protein-restricted mice. J. Endocrinol..

[10] Fan X., Chen D., Wang Y., Tan Y., Zhao H., Zeng J., Li Y., Guo X., Qiu H., Gu Y. (2022). Light intensity alters the effects of light-induced circadian disruption on glucose and lipid metabolism in mice. Am. J. Physiol. Endocrinol. Metab..

[11] Bourgin P., Hubbard J. (2016). Alerting or somnogenic light: Pick your color. PLoS Biol..

[12] Rumanova V.S., Okuliarova M., Zeman M. (2020). Differential effects of constant light and dim light at night on the circadian control of metabolism and hehavior. Int. J. Mol. Sci..

[13] Opperhuizen A.L., Stenvers D.J., Jansen R.D., Foppen E., Fliers E., Kalsbeek A. (2017). Light at night acutely impairs glucose tolerance in a time-, intensity- and wavelength-dependent manner in rats. Diabetologia.

[14] Kwaifa I.K., Bahari H., Yong Y.K., Noor S.M. (2020). Endothelial dysfunction in obesity-induced inflammation: Molecular mechanisms and clinical implications. Biomolecules.

[15] Kawai T., Autieri M.V., Scalia R. (2021). Adipose tissue inflammation and metabolic dysfunction in obesity. Am. J. Physiol. Cell. Physiol..

[16] Shimizu I., Walsh K. (2015). The Whitening of brown fat and its implications for weight management in obesity. Curr. Obes. Rep..

[17] Xu P., Wang J., Hong F., Wang S., Jin X., Xue T., Jia L., Zhai Y. (2017). Melatonin prevents obesity through modulation of gut microbiota in mice. J. Pineal Res..

[18] Fonken L., Nelson R. (2014). The effects of light at night on circadian clocks and metabolism. Endocr. Rev..

[19] Potter G.D., Skene D.J., Arendt J., Cade J.E., Grant P.J., Hardie L.J. (2016). Circadian rhythm and sleep disruption: Causes, metabolic consequences, and countermeasures. Endocr. Rev..

[20] Kiehn J.T., Tsang A.H., Heyde I., Leinweber B., Kolbe I., Leliavski A., Oster H. (2017). Circadian rhythms in adipose tissue physiology. Compr. Physiol..

[21] Froy O., Garaulet M. (2018). The Circadian clock in white and brown adipose tissue: Mechanistic, endocrine, and clinical aspects. Endocr. Rev..

[22] Guan Q., Wang Z., Cao J., Dong Y., Chen Y. (2022). Monochromatic blue light not green light exposure is associated with continuous light-induced hepatic steatosis in high fat diet fed-mice via oxidative stress. Ecotoxicol. Environ. Saf..

[23] Koo Y.S., Song J.Y., Joo E.Y., Lee H.J., Lee E., Lee S.K., Jung K.Y. (2016). Outdoor artificial light at night, obesity, and sleep health: Cross-sectional analysis in the KoGES study. Chronobiol. Int..

[24] Masís-Vargas A., Hicks D., Kalsbeek A., Mendoza J. (2019). Blue light at night acutely impairs glucose tolerance and increases sugar intake in the diurnal rodent Arvicanthis ansorgei in a sex-dependent manner. Physiol. Rep..

[25] Hong F., Pan S., Xu P., Xue T., Wang J., Guo Y., Jia L., Qiao X., Li L., Zhai Y. (2020). Melatonin orchestrates lipid homeostasis through the hepatointestinal circadian clock and microbiota during constant light exposure. Cells.

[26] Nagai N., Ayaki M., Yanagawa T., Hattori A., Negishi K., Mori T., Nakamura T.J., Tsubota K. (2019). Suppression of Blue Light at Night Ameliorates Metabolic Abnormalities by Controlling Circadian Rhythms. Investig. Ophthalmol. Vis. Sci..

[27] Zhang S., Xu M., Shen Z., Shang C., Zhang W., Chen S., Liu C. (2021). Green light exposure aggravates high-fat diet feeding-induced hepatic steatosis and pancreatic dysfunction in male mice. Ecotoxicol. Environ. Saf..

[28] Canbay A., Bechmann L., Gerken G. (2007). Lipid metabolism in the liver. Z. Für Gastroenterol..

[29] Gooley J.J. (2016). Circadian regulation of lipid metabolism. Proc. Nutr. Soc..

[30] Turek F.W., Joshu C., Kohsaka A., Lin E., Ivanova G., McDearmon E., Laposky A., Losee-Olson S., Easton A., Jensen D.R. (2005). Obesity and metabolic syndrome in circadian Clock mutant mice. Science.

[31] Shimba S., Ogawa T., Hitosugi S., Ichihashi Y., Nakadaira Y., Kobayashi M., Tezuka M., Kosuge Y., Ishige K., Ito Y. (2011). Deficient of a clock gene, brain and muscle Arnt-like protein-1 (BMAL1), induces dyslipidemia and ectopic fat formation. PLoS ONE.

[32] Kennaway D.J., Varcoe T.J., Voultsios A., Boden M.J. (2013). Global loss of bmal1 expression alters adipose tissue hormones, gene expression and glucose metabolism. PLoS ONE.

[33] Ribas-Latre A., Eckel-Mahan K. (2022). Nutrients and the circadian clock: A partnership controlling adipose tissue function and health. Nutrients.

[34] Wahl S., Engelhardt M., Schaupp P., Lappe C., Ivanov I.V. (2019). The inner clock-Blue light sets the human rhythm. J. Biophotonics.

[35] Martin J.S., Laberge L., Sasseville A., Bérubé M., Alain S., Lavoie J., Houle J., Hébert M. (2021). Timely use of in-car dim blue light and blue blockers in the morning does not improve circadian adaptation of fast rotating shift workers. Chronobiol. Int..

[36] Sage D., Ganem J., Guillaumond F., Laforge-Anglade G., François-Bellan A.M., Bosler O., Becquet D. (2004). Influence of the corticosterone rhythm on photic entrainment of locomotor activity in rats. J. Biol. Rhythm..

